# Adsorption and Recognition Property of Tyrosine Molecularly Imprinted Polymer Prepared via Electron Beam Irradiation

**DOI:** 10.3390/polym15204048

**Published:** 2023-10-11

**Authors:** Pengpai Miao, Yanan Sang, Jie Gao, Xiaobing Han, Yuan Zhao, Tao Chen

**Affiliations:** Hubei Key Laboratory of Radiation Chemistry and Functional Materials, School of Pharmacy, School of Nuclear Technology and Chemistry & Biology, Hubei University of Science and Technology, Xianning 437100, China; zzu666666@126.com (P.M.); sangyanan2021@163.com (Y.S.); hanxiaobing@hbust.edu.cn (X.H.); zhyf308@hbust.edu.cn (Y.Z.)

**Keywords:** molecularly imprinted polymer, electron beam, L-tyrosine, adsorption, recognition

## Abstract

To realize the selective separation of L-tyrosine (L-Tyr) and avoid the drawbacks of traditional thermal polymerization, electron beam irradiation polymerization was developed for the fabrication of L-Tyr molecularly imprinted polymers (MIPs). Firstly, L-Tyr MIPs were prepared with methacrylic acid and ethylene glycol dimethacrylate and without an initiator. Then, the influence of absorbed dosage and temperature on the adsorption capacity of L-Tyr, as well as the thermodynamic behavior, were investigated. The maximum adsorption capacity of 10.96 mg/g for MIPs was obtained with an irradiation dosage of 340 kGy under 15 °C, and the *ΔH^0^* and *ΔS^0^* of the adsorption process are −99.79 kJ/mol and −0.31 kJ/mol·K, respectively. In addition, the effect of adsorption time on adsorption performance was evaluated under different initial concentrations, and the kinetic behavior was fitted with four different models. Finally, the recognition property of the obtained MIPs was investigated with L-Tyr and two analogues. The obtained MIPs have an imprinting factor of 5.1 and relatively high selective coefficients of 3.9 and 3.5 against L-tryptophan and L-phenylalanine, respectively. This work not only provided an L-Tyr MIP with high adsorption capacity and selectivity but also provided an effective and clean method for the synthesis of MIPs.

## 1. Introduction

L-Tyr is a valuable amino acid, which has been widely used for the improvement of mental alertness and memory [1]. In addition, L-Tyr is an important intermediate for drugs in the field of pharmaceutical areas, such as adrenaline, norepinephrine, and dopamine [2]. Now, L-Tyr is mainly obtained through three different methods, including extraction from the mixture of hydrolyzed protein, biocatalytic synthesis with tyrosine lyase, and fermentation with microbial [3]. To obtain pure L-Tyr product, the produced L-Tyr needs effective separation from the production mixture. Traditional separation and purification methods of amino acids include precipitation, extraction, ion exchange, etc. [4]. However, a large number of reagents and solvents, as well as a lot of time, are needed for these methods. In addition, L-Tyr with high-content impurities is always obtained with these methods, as they lack selectivity [5,6]. As mentioned above, selective separation of L-Tyr is highly desirable for production, detection, and application.

Molecularly imprinted polymers (MIPs) are porous matter fabricated via imprinting technology, which has perfect selectivity for the templates in spatial structure and binding sites [7]. According to the location of templates distributed into the imprinted polymers, the MIPs can be classified as traditional bulk MIPs, novel surface MIPs, and epitope MIPs [8]. Bulk MIPs are the first-generation products with imprinting technology, which are prepared with template-functional monomer complex, cross-linkers, and initiators. For the bulk MIPs, the template is evenly distributed into the polymeric matrix, leading to the hard removal of template locates in the center. To address this issue, surface MIPs are developed with different carriers, and the template is only located at the surface layer. For further improvement of the removal efficiency and recognition speed of the template, especially for biomacromolecules such as peptides and proteins, epitope MIPs were developed. The structure of epitope MIPs is similar to that of surface MIPs, only the exposed chain segments rather than the whole biomacromolecule were selected as the template. The MIPs were mainly fabricated through radical polymerization, with cross-linking agents, functional monomers, and initiators [9]. Due to its excellent recognition ability and wide applicability, MIPs have been successfully applied in the field of sample preparation, chemical sensors, controlled release, and separation of enantiomers [10].

Now, MIPs have also been applied for the selective separation and detection of L-Tyr [11,12]. The first reported L-Tyr MIPs were imprinted polymer beads, which were prepared via suspension polymerization with styrene and different functional monomers. The highest adsorption capacity (0.276 mmol/g) was obtained for the MIPs prepared with functional monomer of 2-acrylamido-2-methylpropanesulfonic acid. In addition, when the competitive molecule is phenylalanine, a large separation factor of 1.93 was obtained [13]. Bio-based chitosan-gelatin molecularly imprinted membranes have also been prepared for the separation of L-Tyr, with a porogen of polyethylene glycol. The obtained imprinted membrane has a relatively low swelling rate of 9.55% and a larger permeability of L-Tyr than 5-sulfosalicylic acid [14]. Mesoporous MIPs of L-Tyr with high surface area were prepared through solution polymerization and used in the controlled release of L-Tyr. The obtained MIP exhibited a high load capacity of 253 mg/g, and the release behavior obeys the zero-order model [15]. The imprinted chromogenic membrane of L-Tyr was fabricated with electrospinning technology and used for the recognition and visualized detection of L-Tyr. The obtained membrane exhibits a high imprinting factor of 2.65 and visible color change for the recognition and detection of L-Tyr [16]. Imprinted cryogel composite was also prepared for the selective adsorption of L-Tyr, wherein the highest adsorption capacity reached 62.27 mg/g and high selectivity was observed among three different amino acids. In addition, after eight adsorption-desorption recycles, no obvious change was observed in the performance of imprinted cryogel [17]. Though L-Tyr MIPs have been developed for their selective separation and detection, all of them are fabricated with traditional thermal polymerization, which suffers from the issues of time consumption, environment pollution, and initiator residue.

Novel irradiation polymerization was developed for the fabrication of MIPs, especially for the electron beam (EB) irradiation polymerization, which not only shortens the preparation time, but also avoids environmental pollution and initiator residue [8,9]. MIPs of sulfamethazine were prepared by EB irradiation polymerization [18], wherein the optimal irradiation dose was found to be 150 kGy, the maximum adsorption capacity of sulfamethazine was 31 mg/g, and the imprinting factor was as high as 12.91. Molecularly imprinted nanospheres of chloramphenicol were fabricated with precipitation polymerization under EB irradiation [19], and the obtained nanospheres have a large specific area of 280.25 m^2^/g. The maximum adsorption capacity of chloramphenicol reached 107.65 µmol/g, and the nanospheres showed a higher selectivity of chloramphenicol than thiampenicol and naproxen. Baicalein-based MIPs were also prepared with EB irradiation polymerization [20], wherein infrared spectrometry demonstrated the successful fabrication of MIPs and the scanning electron microscopy image of the obtained MIPs exhibited an irregular network. However, there are no reports about the preparation of L-Tyr MIPs with EB radiation polymerization.

Thus, the effective and clean EB radiation polymerization was developed for the fabrication of L-Tyr MIPs in this work. Then, the obtained L-Tyr MIPs were characterized by a series of techniques, and adsorption performance was determined under different conditions. Finally, the adsorption mechanism and recognition property were also investigated.

## 2. Materials and Methods

### 2.1. Materials

L-tyrosine (L-Tyr, ≥99%), L-phenylalanine (L-Phe, ≥99%), L-tryptophan (L-Trp, ≥99%), methacrylic acid (MAA, AR), acetonitrile, methanol, and acetic acid were purchased from Sinopharm Chemical Reagent Co., Ltd. (Shanghai, China). Ethylene glycol dimethacrylate (EGDMA, ≥99%) was provided from HWRK Chemical Co. Ltd. (Beijing, China). All reagents and solvents were used without further purification, and other reagents and solvents were of AR grade.

### 2.2. Fabrication of L-Tyr MIPs

**Figure 1:**
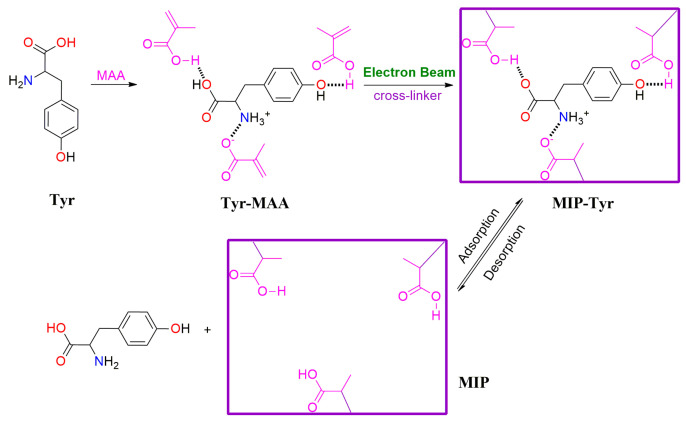
The preparation procedure of L-Tyr MIPs via EB radiation polymerization.

The fabrication procedure is shown in Figure 1 [18,19,20]: l mmol L-tyrosine and 4 mmol methacrylic acid were added into 20 mL acetonitrile and stirred for 2 h to form template-functional monomer complexes (Tyr-MAA). Then, 20 mmol ethylene glycol dimethacrylate was added into the mixture, and bubbling nitrogen was added to remove oxygen for 30 min. After being sealed with polyethylene bags, five samples with the same composition were irradiated via an electron accelerator (1 MeV, Wasik Associates, Dracut, MA, USA) with 260, 300, 340, 380, or 420 kGy. With the EB irradiation, the liquid mixture changed to solid product, and the solid product was washed with methanol and centrifuged to remove the unreacted raw materials. Then, the obtained materials (MIP-Tyr) were washed with methanol/water/acetic acid (20/50/30) under stirring and sonication to extract the template until no L-tyrosine was detected from the eluent by UV spectroscopy (234 nm). For comparison, non-molecularly imprinted polymer (NIP) was fabricated with the same method, only without adding the template of L-tyrosine.

### 2.3. Characterization

Morphology of the fabricated imprinted materials was determined by scanning electron microscopy under 10 kV (SEM, ZEISS Gemini 300, Jena, Germany). The chemical composition of the obtained samples was determined with Fourier transform infrared spectroscopy (FITR, Avatar 360 Nicolet instrument, Markham, ON, Canada). The crystal structure of imprinted materials was demonstrated by X-ray powder diffraction (XRD, LabX 6100, Shimadzu, Tokyo, Japan). The thermogravimetry (TG) analysis was conducted via a TG 209F3 instrument (NETZSCH Scientific Instruments Ltd., Shanghai, China) under N2 atmosphere with a heating rate of 10 °C/min. The absorbance of the L-Tyr solution was determined with UV-vis spectroscopy (UV-1000, Shanghaiaoyi, Shanghai, China).

### 2.4. Adsorption and Recognition Experiment

#### 2.4.1. Effect of Irradiation and Temperature

According to the UV-vis spectrum of the L-Tyr solution, the maximum adsorption wavelength was observed at 234 nm [14]. Then, a series of L-Tyr solutions (0.5, 1, 2, 4, 6, 8, 10 mg/L) were prepared to calibrate the standard curve. The obtained standard curve is A = 0.05322 C- 0.00664, with a coefficient R^2^ = 0.9997.

To reveal the influence of irradiation dosage on the adsorption performance of L-Tyr, batch experiments were conducted. Typically [15], 10 mg MIPs are added into 10 mL 400 mg/L L-Tyr solution and the solution is placed in a bath shaker at 25 °C for 24 h. The batch experiments of different solution temperature were conducted at the same condition for 8 h, and all of the adsorption experiments were repeated three times. The adsorption capacity Q (mg/g) of MIPs was calculated following Equation (1):

Q = (*C_0_*–*C_t_*) × *V*/*m* (1)

where *C_0_* and *C_t_* are the initial concentration and equilibrium concentration, respectively, while *V* (mL) and *m* (mg) stand for the volume of solution and weight of MIPs.

Based on the result of adsorption at different temperatures, thermodynamic parameters of *ΔG^0^* (Gibbs free energy change), *ΔH^0^* (enthalpy change), and *ΔS^0^* (entropy change) were calculated by Equations (2–4) [21]:

*K* = Q_e_/C_e_ (2)

*ΔG^0^* = −RT ln *K* (3)

*ΔG^0^* = *ΔH^0^* − *T* × *ΔS^0^* (4)

where *Q_e_* and *C_e_* are the adsorption capacity and equilibrium concentration, respectively, and *R* is 8.314 J/mol·K. The *ΔH^0^* and *ΔS^0^* are calculated from the linear plot of ln*K* versus 1/*T*.

#### 2.4.2. Adsorption Kinetics

The influence of adsorption time on the capacity was evaluated with the concentrations of 200, 300, and 400 mg/L. The kinetics were investigated with predetermined intervals within 0–5 h. Pseudo-first-order (PFO), pseudo-second-order (PSO), Elovich, and Weber–Morris (intraparticles diffusion, IPD) kinetics models were used to assess the kinetic process, and corresponding kinetic parameters were calculated by Equations (5)–(8) [16,22,23,24]:

(5) $$\log(Qe-Qt)=\log Qe-k_{1}t$$

(6) $$\frac{t}{Qt}=\frac{1}{k_{2}Q_{e}^{2}}+\frac{t}{Qe}$$

(7) $$Qt=\frac{\ln\alpha \beta }{\beta }+\frac{1}{\beta }\ln t$$

(8) $$Qt=kpt^{1/2}+C$$

where *Q_e_* and *Q_t_* are the adsorption capacity at equilibrium and time *t*, respectively, *k*_1_, *k*_2_, k_p_ are the rate constants of the PFO, PSO, and IPD models, *α* is the initial adsorption rate, *β* is the desorption constant, and *C* is a constant.

#### 2.4.3. Specific Adsorption

Two kinds of L-Tyr analogues were used as contrasts, namely L-phenylalanine (L-Phe) and L-tryptophan (L-Trp). Solutions of 400 mg/L L-Tyr and analogues were prepared under the same conditions, 10 mg MIPs were added into 10 mL solution, and the adsorptions were conducted at 25 °C for 8 h. For comparison, NIP was also used for the adsorption of L-Tyr and analogues in the same condition. The selective coefficient α and imprinting factor β are calculated as following Equations (9) and (10) [13,16,17]:

*α* = *Q_L-Tyr_/Q_other_* (9)

*β* = *Q_MIP_/Q_NIP_* (10)

where *Q_L-Tyr_* and *Q_other_* are the adsorption capacity toward L-Tyr and analogues and *Q_MIP_* and *Q_NIP_* are the adsorption capacity of MIPs and NIP.

## 3. Results

### 3.1. Morphology Analysis

**Figure 2:**
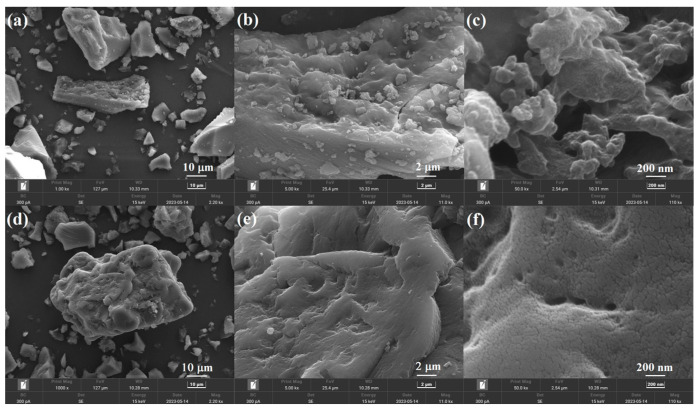
SEM images of L-Tyr MIP (**a**–**c**) and NIP (**d**–**f**) with different resolutions.

The morphology of the L-Tyr MIP and NIP were revealed by SEM and are shown in Figure 2. As shown in Figure 2a,d, the MIP and NIP are particles, and the NIP has a larger size than that of MIPs. Compared with the high-resolution image of NIP (Figure 2e,f), a rougher surface and many cavities were observed for images of MIP, which are beneficial for the adsorption and recognition of L-Tyr. The formation of a rough surface and porous structure can be assigned to the different preparation processes of MIP and NIP [11,14], template-functional monomer complexes were formed in the fabrication of MIP, and a porous structure appeared after the removal of the template. In addition, the formed cavities are complementary in binding sites, shape, and size to the template.

### 3.2. FTIR Analysis

**Figure 3:**
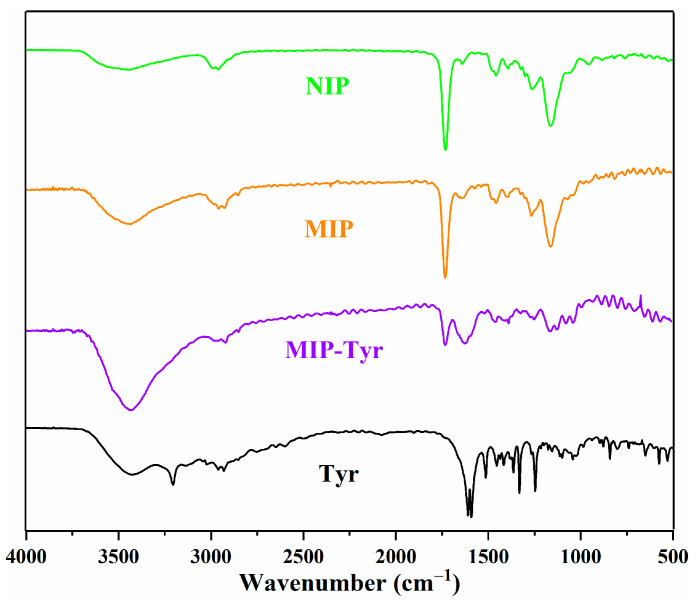
FTIR spectra of L-Tyr, MIP-Tyr, MIP, and NIP.

The chemical structure of L-Tyr, MIP-Tyr, MIP, and NIP were demonstrated with FTIR spectroscopy and are presented in Figure 3. For the spectra of L-Tyr, a broad peak from 3300 to 3600 cm^−1^ belonged to the vibration of -OH (carboxylic acid and phenol hydroxyl), and 3207 cm^−1^ was the vibration peak of -NH_2_. In addition, 2965 and 2967 cm^−1^ belonged to the C-H vibration of the aliphatic chain, and peaks at 1620 and 1588 cm^−1^ were assigned to the antisymmetric and symmetric vibrations of C=O. Values of 1517, 1457, and 1420 cm^−1^ were assigned to the skeleton vibration of the aromatic ring [25,26]. For the MIP-Tyr, 3431 cm^−1^ was assigned to the vibration of -OH, and 1728 cm^−1^ was assigned to the C=O originating from the functional monomer MAA and cross-linker EGDMA. An obvious C=O vibration peak for L-Tyr was also observed at 1623 cm^−1^, demonstrating the successful fabrication of the L-Tyr-loaded MIP [19,27]. For the spectra of MIP, the C=O vibration of L-Tyr disappeared, revealing the successful removal of the template molecule. In addition, no obvious difference was observed for the obtained NIP and MIP, which further demonstrated the successful preparation of the MIP.

### 3.3. XRD Analysis

**Figure 4:**
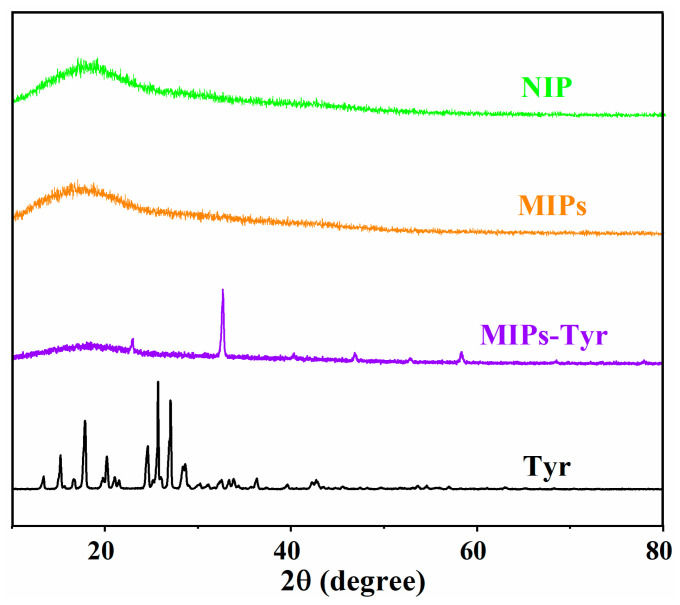
XRD patterns of L-Tyr, MIP-Tyr, MIP, and NIP.

The crystal structure of L-Tyr, MIP-Tyr, MIP, and NIP were demonstrated with XRD spectroscopy and are presented in Figure 4. As shown in Figure 4 sharp peaks at 15.2°, 17.9°, 20.1°, 24.5°, 25.7°, 27.0°, and 28.6° suggest the crystal structure of L-Tyr [28,29]. For the MIP-Tyr, only one sharp peak at 32.7° was observed, indicating the decrease of the crystalline degree of L-Tyr and the uniform dispersion of L-Tyr into the polymeric matrix [30]. For the XRD patterns of MIP and NIP, only broad peaks were observed at 17.1° and 17.6°, revealing the amorphous structure of polymeric materials [31]. In addition, the disappearance of the sharp peak also demonstrated the successful removal of the template molecule.

### 3.4. TG Analysis

**Figure 5:**
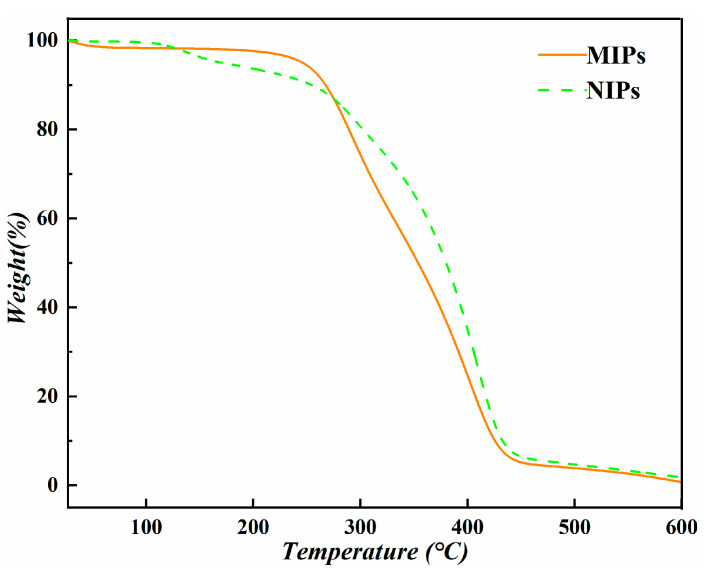
TG curves of MIP and NIP.

The thermal degradation behavior of MIPs and NIPs was revealed by thermogravimetry analysis, as shown in Figure 5, where three major weight loss stages were observed for both MIPs and NIPs. For the MIPs, the small weight loss of 1.7% between 27 and 90 °C is due to the loss of physisorbed water. The obvious weight loss of 92.7% between 190 and 450 °C can be ascribed to the degradation of the cross-linker segment, and the third stage after 450 °C is attributed to the decomposition of the functional monomer segment [32]. For the NIPs, a higher temperature (110 to 270 °C) was observed for the first degradation stage than that of MIPs, which is consistent with the result reported by Segatelli and co-workers [32]. The decomposition behavior of the second and third stages is similar to that of MIPs, and the NIPs show a higher thermal stability [32,33].

### 3.5. Adsorption and Recognition Analysis

#### 3.5.1. Effect of Irradiation and Temperature

**Figure 6:**
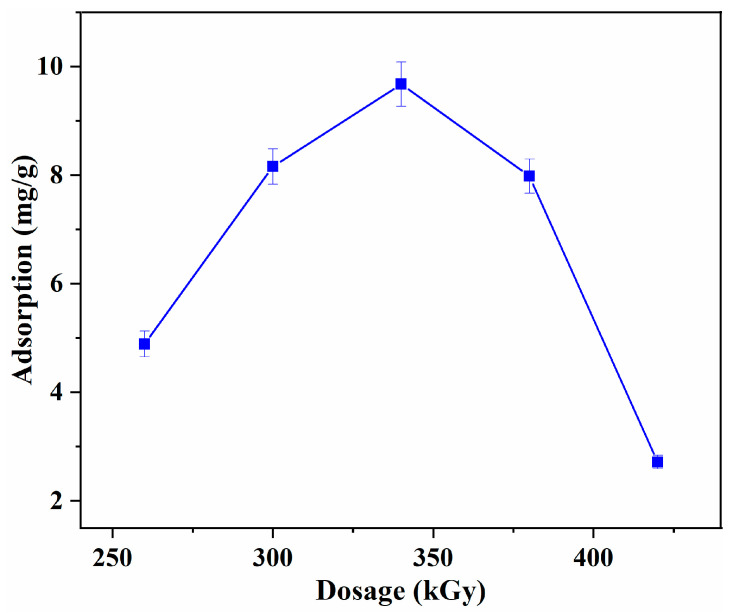
Effect of absorbed dosage on the L-Tyr adsorption capacity.

Since the EB irradiation polymerization obeys the radical polymerization mechanism, the irradiation dosage is the key parameter to the structure and performance of the obtained L-Tyr MIPs because it determines the number of radicals. The influence of absorbed dosage on the adsorption capacity of L-Tyr was investigated first, and the results are presented in Figure 6. With the increase of absorbed dosage, the adsorption capacity increased, and the maximum capacity of 9.67 mg/g was observed at 340 kGy. Further increasing the irradiation dosage leads to the decrease of adsorption capacity, and this can be ascribed to the irradiation dosage having an important influence on the structure of the obtained L-Tyr MIPs. When the irradiation dosage is low, the polymerization rate is very slow and the binding site number in the MIPs is reduced due to the low degree of cross-linking. On the contrary, when the irradiation dosage is high, the polymerization rate is very fast, leading to a high degree of cross-linking and serious blocking of binding sites, which is unfavorable to the mass transfer process, thus greatly reducing the adsorption capacity [18,19]. Hence, the sample obtained at 340 kGy was chosen for further investigation.

**Figure 7:**
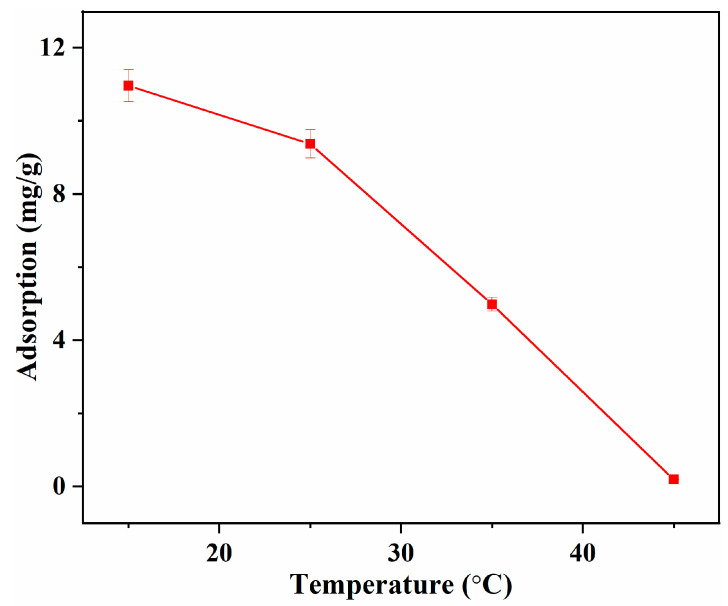
Influence of temperature on the L-Tyr adsorption capacity.

To reveal the energy changes in the adsorption process, the influence of temperature on the adsorption capacity of L-Tyr was investigated, and thermodynamic parameters were calculated according to the result. As presented in Figure 7, the adsorption capacity decreased dramatically with the increase of temperature, indicating the exothermic nature of the process [34]. The maximum capacity of 10.96 mg/g was obtained at 15 °C, which is just a little higher than the adsorption capacity at 25 °C. Though low temperature is favorable for the improvement of adsorption capacity, taking the accessibility of real operation and energy consumption into account, the following investigation was conducted at 25 °C.

**Figure 8:**
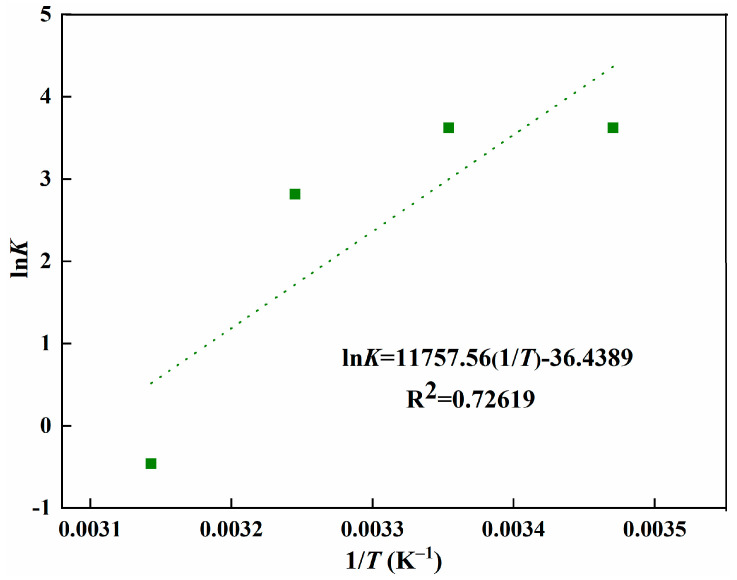
Ln*K* versus 1/*T* for MIPs.

Based on the results of temperature effect on the adsorption, thermodynamic parameters of *ΔG^0^*, *ΔH^0^*, and *ΔS^0^* were calculated according to Equations (2)–(4) [21,34,35,36]. As shown in Figure 8, *ΔH^0^* and *ΔS^0^* were calculated from the linear plot of ln*K* versus 1/*T*, and all of the calculated results are listed in Table 1.

As shown in Table 1, a negative value of *ΔG^0^* was observed at room temperature, indicating that the adsorption of L-Tyr is spontaneous [35]. With the increase of temperature, the *ΔG^0^* increased gradually, revealing that high temperature will hinder the adsorption process, which is consistent with the experiment result. The value of *ΔG^0^* changes to positive when the temperature increases to 318 K, revealing that the process cannot proceed spontaneously [36]. The value of *ΔH^0^* was found to be negative, which demonstrates the adsorption process is exothermic again. In addition, the negative value of *ΔH^0^* also suggests the interaction of ion-pairing and hydrogen bonding between the L-Tyr and MIP [34,36]. The value of *ΔS^0^* was also found to be negative, indicating the order increase of the system, which is the result of the energetic penalty accompanied by the freezing of the *ΔG^0^* rotor [34,36].

#### 3.5.2. Adsorption Kinetics

**Figure 9:**
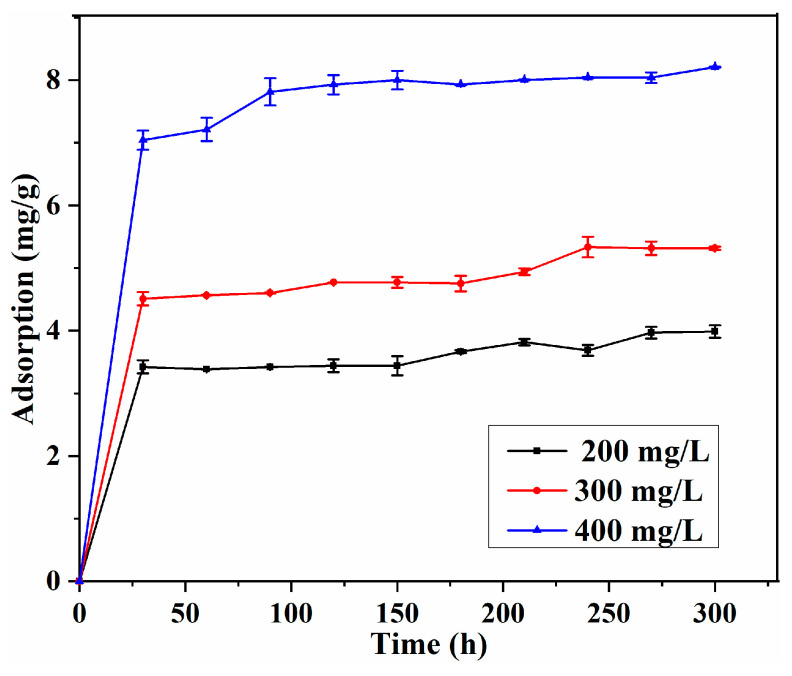
Effect of initial concentration and adsorption time on the adsorption.

The effect of contact time on the capacity was evaluated under different initial concentrations, and the result is presented in Figure 9. The results indicate that the adsorption of L-Tyr with an MIP is very fast, within 0.5 h, and higher capacity was observed for high initial concentration at adsorption equilibrium. In addition, a longer time was needed to reach adsorption equilibrium for the high concentration, demonstrating that the adsorption process is dependent on concentration [37,38].

**Figure 10:**
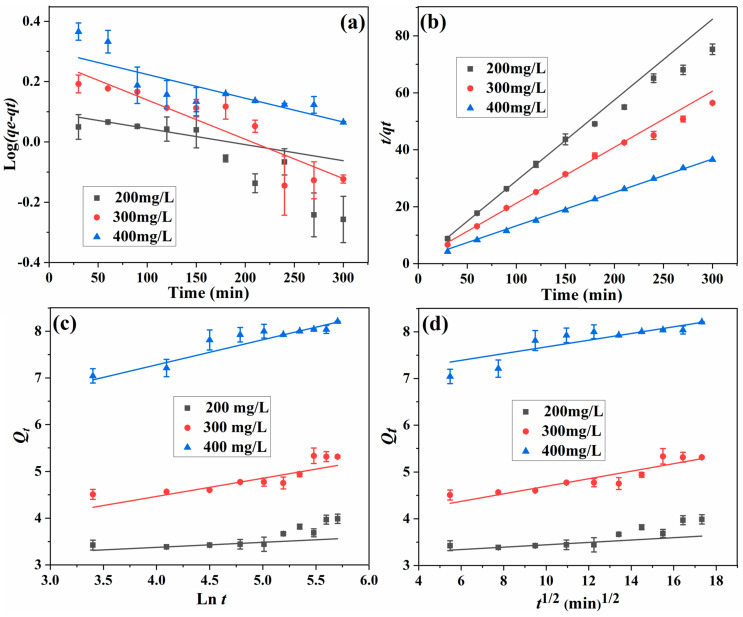
Fitting of PFO (**a**), PSO (**b**), Elovich (**c**), and IPD (**d**) models for the adsorption process.

To reveal the kinetic behavior, the adsorption behavior was fitted with pseudo-first-order (PFO), pseudo-second-order (PSO), Elovich, and Weber–Morris (IPD) kinetic models [16,22,23,24], and the fitting curves are shown in Figure 10. According to Equations (5–8), kinetic parameters are calculated and listed in Table 2.

As shown in Table 2, the value of coefficient R^2^ is in the order of PSO (0.9987–0.9992) > PFO (0.8735–0.9685) > IPD (0.8250–0.9774) > Elovich (0.7688–0.9770). The experimental adsorption capacity is very close to the calculated one for PSO, and the highest coefficient R^2^ was observed for the PSO model. According to the reported literature [39,40], the PSO kinetic model fits well with the adsorption process of the MIP, and the rate-limiting step is chemical adsorption. However, according to the investigation of Simonin, PFO and PSO models are not so suitable for the fitting of the result obtained in this work, as much of the adsorption data in Figure 9 were too close to equilibrium [41]. For the PFO model, when *Q_t_* approaches *Q_e_*, the value of (*Q_e_*–*Q_t_*) becomes smaller and smaller, leading to the accuracy reduction of *k_1_*. In addition, taking much data at equilibrium into account in the PSO kinetic model study is not coherent. As the fitting of Elovich and IPD kinetic models do not involve the data at equilibrium, and the coefficient R^2^ of the IPD model is larger than that of the Elovich model, the IPD is predominant for L-Tyr adsorption, and the process was controlled by diffusion.

#### 3.5.3. Specific Adsorption

**Figure 11:**
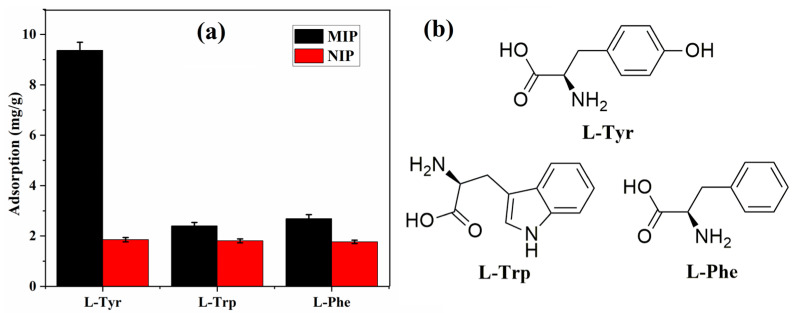
Adsorption capacity (**a**) and molecule structure (**b**) of L-Tyr and analogues.

To reveal the selectivity or recognition property of the obtained MIP, adsorption experiments were conducted with L-Tyr and analogues (L-Trp, L-Phe). As shown in Figure 11a, the maximum capacity of L-Tyr is much higher than those of L-Trp (2.40 mg/g) and L-Phe (2.68 mg/g), which indicates that the formed cavity in the L-Tyr MIP can especially recognize L-Tyr [13,42]. Although the analogues have a similar structure and functional groups with L-Tyr (Figure 11b), the molecular size is different, which makes the difference in adsorption capacity.

Detailed data of selectivity were calculated according to Equations (9) and (10), and the obtained selective coefficient α and imprinting factor β are presented in Table 3. The selective coefficients of L-Tyr are calculated as 3.9 and 3.5 against L-Trp and L-Phe, respectively. In addition, the imprinting factor of L-Tyr is 5.1, which is much higher than those of L-Trp and L-Phe. These results demonstrated that the obtained MIP exhibits a better recognition ability toward the L-Tyr molecule [16,17].

#### 3.5.4. Regeneration Property

**Figure 12:**
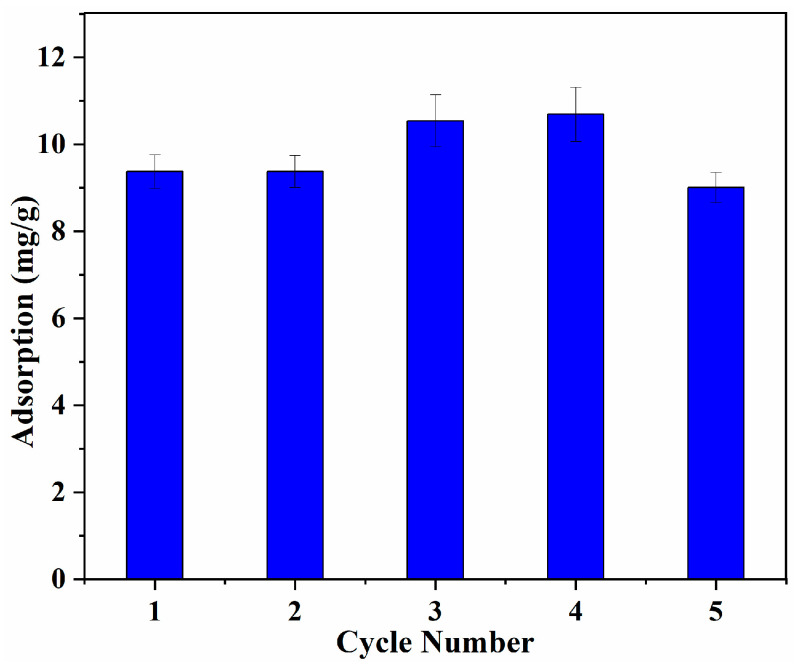
Influence of temperature on the L-Tyr adsorption capacity.

The regeneration property of MIPs is crucial in practical industrial application. The reusability and the stability of the obtained MIP was determined by measuring the adsorption capacity of the MIP for L-Tyr repeatedly after each adsorption-desorption cycle under the same conditions [38,40]. The adsorption capacity of the MIP, which was reused five times, is shown in Figure 12, which demonstrates that the obtained MIP has excellent stability with a negligible decrease in adsorption capacity. The adsorption efficiency only lost about 4% after five adsorption-desorption cycles, which shows a promising application in L-Tyr separation [12,16].

## 4. Conclusions

In summary, an L-Tyr MIP was successfully fabricated via an effective and clean EB irradiation technique. The obtained L-Tyr MIP has a porous structure and a relatively high adsorption capacity of 10.96 mg/g at 15 °C. Thermodynamic investigation indicates that the recognition process is exothermic, and the kinetic parameters reveal that the adsorption process was controlled by diffusion. In addition, the obtained MIP exhibits a higher recognition ability against analogues. Future work will focus on the scale production of MIPs with EB irradiation polymerization and the treatment of complicated samples containing L-Tyr.

## Figures and Tables

**Table 1:** Thermodynamic results for the adsorption of L-Tyr.

Temperature (K)	*ΔG^0^* (kJ/mol)	*ΔH^0^* (kJ/mol)	*ΔS^0^* (kJ/mol·k)
288	−8.67	−99.79	−0.31
298	−8.54		
308	−7.21		
318	1.22		

**Table 2:** Kinetic results of two models for L-Tyr adsorption.

Kinetic Models	Coefficients	200 mg/L	300 mg/L	400 mg/L
PFO	*q*_e,cal_ (mg/g)	1.253	1.861	2.008
	*k*_1_ (min^−1^)	5.34 × 10^−4^	1.3 × 10^−3^	7.89 × 10^−4^
	*R* ^2^	0.8735	0.9320	0.9685
PSO	*q*_e,cal_ (mg/g)	3.522	5.080	8.518
	*k*_2_ (g/mg min)	9.21 × 10^−2^	2.30 × 10^−2^	9.63 × 10^−3^
	*R* ^2^	0.9993	0.9987	0.9992
Elovich	*q*_e,cal_ (mg/g)	3.560	3.524	5.7411
	*α* (mg/g min)	7.47 × 10^10^	6.83 × 10^2^	7.29 × 10^3^
	*β* (g/min)	9.2601	2.5685	1.8562
	*R* ^2^	0.7688	0.8756	0.9770
IPD	*q*_e,cal_ (mg/g)	3.628	5.285	8.204
	*k_p_* (g/kg min^1/2^)	0.0253	0.0806	0.0719
	*C* (g/kg)	3.1896	3.8886	6.9572
	*R* ^2^	0.8250	0.9181	0.9774

**Table 3:** Selective coefficient α and imprinting factor β of MIPs.

Sample	α	β
L-Tyr	/	5.1
L-Trp	3.9	1.3
L-Phe	3.5	1.5

## Data Availability

Data produced in this study can be made available upon a reasonable request.
